# Detecting false-positive disease references in veterinary clinical notes without manual annotations

**DOI:** 10.1038/s41746-019-0108-y

**Published:** 2019-05-03

**Authors:** Noel Kennedy, Dave C. Brodbelt, David B. Church, Dan G. O’Neill

**Affiliations:** 1 IT Department, The Royal Veterinary College, 4 Royal College St, London, NW1 0TU UK; 2 Pathobiology and Population Science, The Royal Veterinary College, Hawkshead Lane, North Mymms, Hatfield, Herts AL9 7TA UK; 3 Clinical Sciences and Services, The Royal Veterinary College, Hawkshead Lane, North Mymms, Hatfield, Herts AL9 7TA UK

**Keywords:** Epidemiology, Diagnosis, Signs and symptoms, Epidemiology, Diagnosis

## Abstract

Clinicians often include references to diseases in clinical notes, which have not been diagnosed in their patients. For some diseases terms, the majority of disease references written in the patient notes may not refer to true disease diagnosis. These references occur because clinicians often use their clinical notes to speculate about disease existence (differential diagnosis) or to state that the disease has been ruled out. To train classifiers for disambiguating disease references, previous researchers built training sets by manually annotating sentences. We show how to create very large training sets without the need for manual annotation. We obtain state-of- the-art classification performance with a bidirectional long short-term memory model trained to distinguish disease references between patients with or without the disease diagnosis in veterinary clinical notes.

## Introduction

Disease references in clinical notes are often negated (“ruled out pancreatitis”), hypothetical (“at risk of developing pancreatitis”), generic (“pancreatitis is more common in males”), historical (“previous history of pancreatitis”), refer to another person (“father had pancreatitis”), hedged (“could be pancreatitis”) or part of a differential diagnosis (“ddx: pancreatitis, gastroenteritis or cholecystitis”). These phenomena have varied semantics which defy a simple common category, but since our application is case finding for epidemiological studies, we use the term ‘false positive' (FP) to refer to a disease mention that does not have a concurrent disease diagnosis recorded for the individual. In comparison, an example true-positive (TP) disease reference would be an assertion or inference that the author of the note believes that the patient had the disease in question at that time (“patient has pancreatitis”). FPs are commonplace in clinical text with 39–83% of recorded clinical signs reported as negated,^1^ which is just one phenomenon which comprises FP disease references.

When epidemiologists search for cases for their studies, they often search for relevant clinical codes, or search for keywords in the clinical text. Using keyword searches in clinical text increases sensitivity (recall)^2^ but reduces a positive predictive value (PPV) (precision).^3,4^ Ford et al.^2^ surveyed 67 papers on case finding in electronic medical records (EMR). They found a significant improvement in the median sensitivity when both clinical text and codes are searched (78% median sensitivity for code and text searches compared with 62% for just codes). However, keyword searching on clinical text can result in lower PPV, because these searches retrieve many FP disease references for patients who have the keywords in their notes but who don’t have the disease diagnosed. The PPV value of keyword searches varied depending on the disease. A search for hyperadrenocorticism cases in a population of 210,824 dogs had a PPV of 12%,^4^ whereas a search for patellar luxation had a PPV of 42.7%.^3^ Another study of veterinary data found that 11% of sentences contained negation and 5% contained speculation.^5^ Therefore, after a keyword search, epidemiologists often spend time manually ruling out FP disease references in order to identify only those patients who have the disease they wish to study.

The aim of this study was to reduce the numbers of FP sentences that an epidemiologist has to check when they are case finding using keyword searches in free text. To do this, we wanted to train a classifier, whose input was a sentence containing a reference to a disease, and whose output indicated if the patient had been diagnosed with that disease, i.e. to mark the disease references as either TP or FP.

We make two contributions:

1. We show how to create large-scale datasets for training FP machine-learning classifiers without any need for manual labelling of disease references. Our method yields the largest training corpus of sentences to date for this purpose.
2. We establish that a bidirectional^6^ long short-term memory^7^ model, without any feature engineering, outperforms the previous state-of-the-art classifier in determining whether a disease reference relates to a diagnosed or undiagnosed patient.

FP is a core problem in clinical natural language processing (NLP), and has been studied in clinical text since at least 1994.^8^ There is a substantial body of work on detection and classification of FP phenomena specifically in clinical text.^9^ The widely implemented NeGex^10^ was based on regular expressions. FP phenomena have been the subject of shared challenges in the biomedical literature: BioNLP09^11^ and CoNLL 2010.^12^ There have also been shared challenges on clinical text: the 2010 i2b2/VA challenge^13^ and SEMEVAL 2015 Task 14.^14^ Ours is the second published work using veterinary data. Cheng et al.^5^ manually annotated 1041 documents from veterinary primary-care practices for the purpose of negation and speculation detection. They annotated cue words such as “not”, “possible”, “suspected”, etc., and then annotated the linguistic scope of the negation or speculation. They trained one conditional random field classifier^15^ to detect cue words, and another to identify linguistic scope. They found that they could increase performance by training on a mixture of both veterinary and human clinical data from Bioscope.^16^

There has been comparatively little innovation in methods for creating datasets of sentences for training FP classifiers. The dominant approach taken in the literature has been to create datasets by hand. Human annotators labelled individual disease references in sentences drawn from a sample corpus.^11,12,14,17,18^ The alternatives to the fully manual method were weakly supervised approaches which started with a small seed set of sentences and iteratively grew the training set.^16,19^ The work of Szarvas^16^ on radiological documents is the most closely related to our method, as it leveraged the association of clinical codes and disease references. They observed that strict clinical coding guidelines required that uncertain or hedged diagnoses should not be coded. Therefore, if a document lacks a code for a disease which is mentioned, then the disease reference is an FP.

We show that by using a distant-supervision approach,^20^ it is not necessary to either label sentences manually, or to clinically code each document, or to have strict or consistent coding standards in order to create a dataset for training an FP classifier. Our method is therefore capable of learning from the large historic EMR, even if these datasets were not annotated for this purpose. This is important because FP classifiers trained on one dataset do not perform as well as those trained on in-domain data,^5,21^ with a similar finding on a veterinary disease classification task.^22^ Cheng et al.^5^ showed that a classifier trained to detect negation cues and scope on out-of-domain data in the form of human clinical notes performed similarly to the rule-based NegEx^10^ algorithm. It was only when in-domain VetCompass data were used for training that substantial improvements in performance were observed (an indicative result was that F1 increased from 63.1 to 74.4 by training on in-domain data). Nie et al.^22^ showed that disease classifiers trained on veterinary referral centre data showed a drop in performance when evaluated against veterinary primary-practice data. It is therefore important to be able to generate an in-domain dataset for training FP classifiers. We show how to do this, even if the dataset was not annotated for this purpose.

We propose the following method for generating a training set of sentences for training a classifier. We want a set of sentences *X* containing a disease reference with corresponding labels *Y*, such that *Y*_*i*_ is the label for sentence *X*_*i*_.

We define the terms as follows:

- False positive (FP): a disease reference in a patient’s notes, where that patient had not been diagnosed with the disease at the time the note was written.
- True positive (TP): a disease reference in a patient’s notes, where that patient had been diagnosed with the disease at the time the note was written.
- A diagnosis: a time-stamped clinical code which declares that the patient has the disease referred to in the patient’s notes at a point in time.
- Diagnostic window: a duration of time around the point in time of diagnosis.

**Fig. 1:**
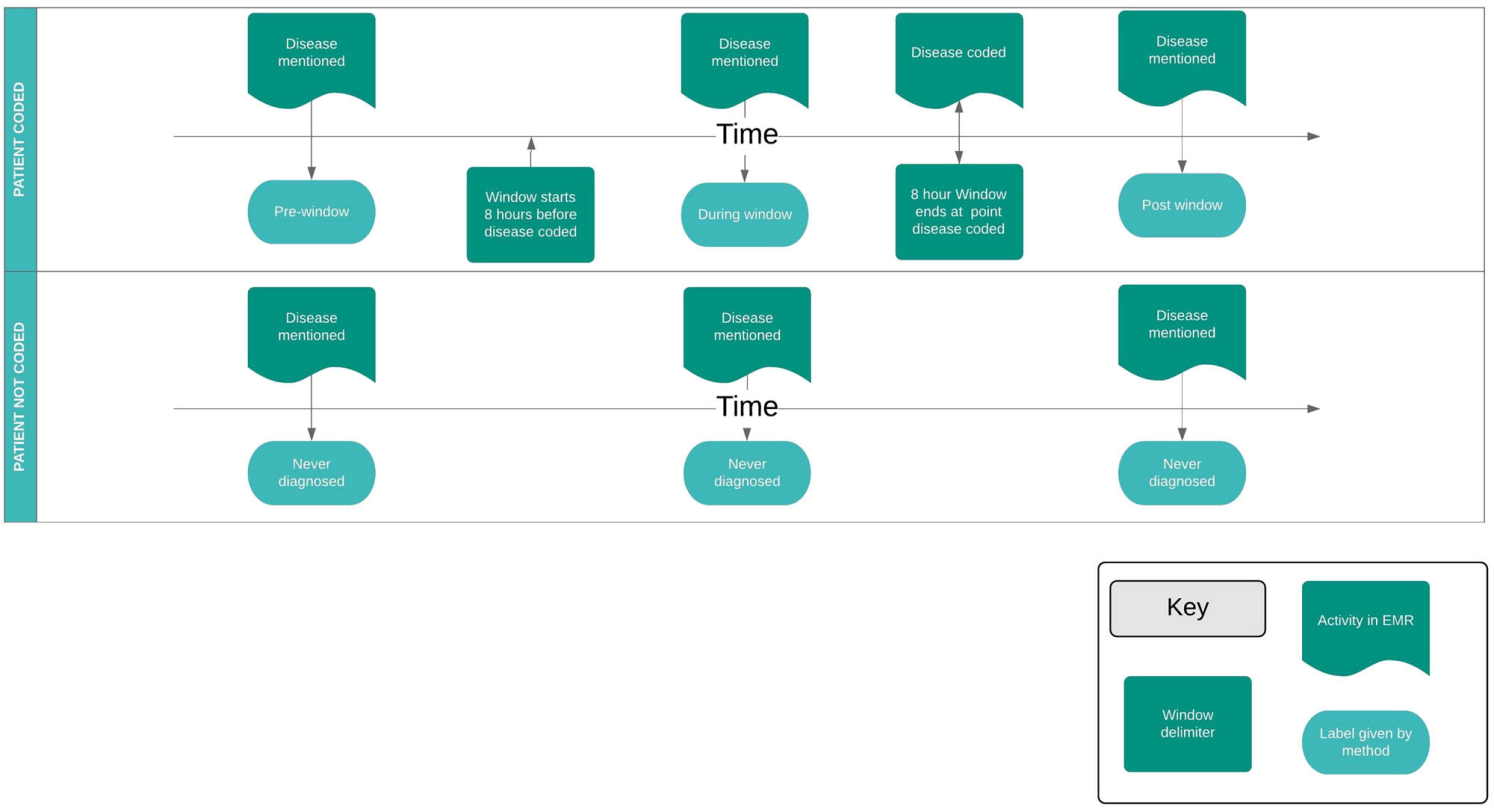
A diagram showing how our method created labels for disease phrases in clinical text. Two patients are represented, one in each row. The patients are treated differently, as one patient received a relevant clinical code, and the other didn't. The bottom row represents a patient that was never coded with the disease that was mentioned in their notes. The area above each row’s timeline represents events in the electronic medical record (EMR) system. The area below the timeline represents interpretations given by our method based on the events in the EMR. Each time the disease is mentioned in the EMR, our method labels the sentence with one of four labels. In the case of the bottom row, where the patient was never coded, the method is simple to apply: all sentences containing disease mentions are given the ‘Never diagnosed’ label. The top patient received a clinical code, indicating that the patient had been diagnosed with the disease which was mentioned in their notes. This row is slightly more complicated, as there are three potential labels that can be applied: ‘Pre-window’, ‘During window’ and ‘After window’. The label applied depends on temporal relationship in the EMR between the disease reference and the clinical code

A 4-class multiclass labelling scheme for disease references was constructed (see Fig. 1). The 4 classes corresponded to TP/FP classification of disease references as follows:

- References to diseases in notes where the patient was never diagnosed with that disease are FP.
- References to diseases in notes written prior to the diagnostic window are FP.
- References to diseases in notes written during the diagnostic window are ambiguous as to their TP/FP status.
- References to diseases in notes written after the diagnostic window are TP.

We trained several classifiers to take a sentence *X*_*i*_ as input, and to output a label *Y*_*i*_ where the label is one of the four classes defined above.

Although we are using coded diagnoses to determine true disease presence, it is important to treat this with caution: coded diagnoses are not always an accurate indicator that the patient has the disease. Often, a diagnosis is coded, but later investigation results in a different diagnosis which should be interpreted as, meaning, the patient never had the first coded disease. Also, although care is taken in the clinic to ensure that all true cases receive a code, some cases will mistakenly not receive a code. All machine-learning methods that treat codes as the gold standard have this limitation, so the issue is not limited to our method.

## Results

### Application of the method and description of classifiers

We applied our method to data drawn from the VetCompass™ corpus.^23^ VetCompass is a veterinary clinical corpus containing electronic medical records (EMR) for 6 million animals at the time of the experiment. The corpus aggregated EMR recorded in hundreds of UK clinics (roughly 10% of all UK veterinary clinics). We applied our method to two subsets of VetCompass patients: those in primary care and those in referral care. This split is equivalent to general practice (GP) vs. hospitalised patients in the medical domain. Separate application was necessary because the mechanism of clinical coding differed between the two data subsets (see the “Discussion” section). We then concatenated the primary-care and referral subsets into a single dataset.

We experimented with a number of different neural models, which produced a fixed-length vector representing the sentence. We wanted to explore which of the neural architectures were better at capturing the syntactic and semantic knowledge necessary for this task. Each neural model ended with a soft-max layer with four units, which represented our four classes. We now describe each of the models we experimented with:

- The CNN model of Zhang and Wallace^24^ henceforth referred to as CNN-flat. This model had multiple layers of convolutions which span different numbers of adjacent tokens. A max-pooling layer took the maximum score each filter obtained anywhere in the sentence and these scores were then concatenated to form a fixed-length vector representing the sentence. This was a relatively flat model because, although it had multiple convolutional layers, these layers were not stacked on each other, but were instead all fed the same input sentence independently and so were all at the same depth in the model.
- The Hierarchical ConvNet model of Conneau et al.^25^ henceforth referred to as CNN-hierarchical. This was also a CNN model but differed from CNN-flat in that it stacked convolutions on the output of previous convolutions. CNN-hierarchical then formed a fixed- length sentence vector by concatenating the representations learned at each level in the convolutional hierarchy.
- A hierarchical CNN similar to CNN-hierarchal henceforth referred to as CNN-funnel. CNN-funnel progressively compressed the sentence representation from the original input token space into smaller and smaller dimension vectors. CNN-funnel had three stacked convolutional layers. The representation compression occurred through two mechanisms: the number of filters in each subsequent convolutional layer had fewer filters than the previous layer. Each convolutional layer was also followed by a max-pooling layer of size 5, which selected only the most relevant local features for each filter.
- A bidirectional LSTM^7^ henceforth referred to as BILSTM. This was not a CNN but a recurrent neural model, which took as input the current token in the sentence, and the previous state of the model. The bidirectional aspect meant that there are two LSTM models: the first read the sentence in order, and the second read in reverse order. The output of each LSTM was concatenated, and then a max-pooling layer took the highest score longitudinally to form a fixed-length sentence vector.
- The baseline classifier was UTH-CCB,^26^ which was the previous state-of-the art classifier, as determined by Task 14 in SemEval-2015.^14^ UTH-CCB was a Support Vector Machine^27^ based algorithm. It had a number of manually engineered features, including N-Grams, context words surrounding the disease reference, a dictionary of FP-indicating words and phrases (e.g. “not”, “could be”) and syntactical dependencies of the disease phrase constituent.

### Evaluation and metrics

Epidemiologists trade-off sensitivity (recall) and specificity in case finding. Presenting classification performance as receiver-operating characteristic (ROC) curves visualises these trade-offs at points on the curve. Reporting classification precision and recall requires us to choose to report a point on the curve, but the final decision on which threshold to select can be left to the epidemiologist to suit their purposes. Area under the ROC curve (AUROC) is a single metric which measures performance at all points on the ROC curve, so we chose this to compare the classifiers. In general, a higher AUROC indicates that the classifier is better, but small differences between classifiers might not be statistically significant. We also calculated the PPV (precision), sensitivity (recall) and F1 score as these metrics are common in the literature.^5,14^

We carried out two experiments to evaluate the models. The first experiment (the seen-disease experiment) was designed to evaluate the models’ ability to classify disease references for the 168 diseases that it had seen in training. For the seen-disease experiment, the dataset *X* was split randomly into training, development and test sets with an 80/10/10 split. All classifiers were trained on the training set. We searched for optimal hyperparameters on the development set and the final evaluation occurred on the test set. See Tables 1 and 2 for the results of the seen-disease experiment, which we discuss later. The second experiment (the unseen-disease experiment) evaluated the models’ ability to classify references to diseases that were unseen in training. We wanted to know how the models would perform on disease references that they had not been trained on, i.e. the generalisability of the models. We created 168 pairs of training and test sets from the dataset *X*; one pair for each of the 168 diseases. For a given disease, if a sentence contained a disease reference to that disease, the sentence was allocated to the test set; all other sentences were allocated to the training set. This meant that the models were evaluated against disease references that were unseen at training time. Each model was trained from a random initial state 168 times, once for each disease. The resulting 168 pairs of true labels and the model’s predictions were then concatenated to calculate the metrics for each model. See Tables 3 and 4 for the results of the unseen-disease experiment, which we discuss later.

**Table 1:** Seen diseases: per-class AUROC classification performance

Classifier	Never diagnosed	Pre-window	Post window	During window
BILSTM	**0.898**	**0.838**	**0.894**	0.943
CNN-flat	0.865	0.800	0.864	0.925
CNN-funnel	0.883	0.823	0.880	0.927
CNN-hierarchical	0.890	0.823	0.886	**0.946**
UTH-CCB (baseline)	0.879	0.803	0.876	0.872

Our method produced a single label from one of 4 classes for each disease reference in patients’ clinical notes. The ‘window’ was the diagnostic window around the point in time of diagnosis. During the window, it is ambiguous if the patient has been diagnosed with the mentioned disease, but for the other three labels, we know if the patient was diagnosed with the disease or not. We trained five models on a dataset produced by our method. This experiment evaluated the models against diseases seen in training, but unseen sentences. The BILSTM obtained the highest ROC AUC scores of most classes. The Never-diagnosed and Post-window classes account for 96% of the sentences, so higher performance on these classes is crucial. The cells in bold formatting indicate the model which obtained the highest score for the class

**Table 2:** Seen diseases: micro and macro average precision, recall and F1

Classifier	Precision (PPV)		Recall (sensitivity)		F1	
	Macro-average	Micro-average	Macro-average	Micro-average	Macro-average	Micro-average
BILSTM	**0.584**	**0.749**	**0.631**	0.845	**0.605**	**0.795**
CNN-flat	0.537	0.697	0.587	0.846	0.555	0.764
CNN-funnel	0.583	0.732	0.597	0.838	0.584	0.782
CNN-hierarchical	0.568	0.729	0.619	**0.855**	0.590	0.786
UTH-CCB (baseline)	0.569	0.731	0.572	0.841	0.555	0.782

These are the precision (positive predictive value), recall (sensitivity) and F1 metrics for the seen- diseases experiment (i.e. the same experiment described in Table 1). The cells in bold indicate the model which obtained the highest score for the metric

**Table 3:** Unseen diseases: per-class AUROC classification performance

Classifier	Never diagnosed	Pre-window	Post window	During window
BILSTM	**0.730**	**0.675**	**0.741**	**0.908**
CNN-flat	0.675	0.639	0.685	0.894
CNN-funnel	0.680	0.633	0.691	0.899
CNN-hierarchical	0.712	0.637	0.722	0.895
UTH-CCB (baseline)	0.709	0.639	0.719	0.842

This experiment used the same dataset produced by our method, but the sentences that were seen at training time were different. In this experiment, the models were evaluated against diseases that the models did not see during training; they were evaluated against held-out diseases. This experiment evaluated the ability of the models to generalise to diseases that were not found in our training set. The cells in bold indicate the model which obtained the highest score for the class

**Table 4:** Unseen diseases: micro and macro average precision, recall and F1

Classifier	Precision (PPV)		Recall (sensitivity)		F1	
	Macro-average	Micro-average	Macro-average	Micro-average	Macro-average	Micro-average
BILSTM	0.454	**0.625**	0.470	0.773	0.422	**0.691**
CNN-flat	0.441	0.581	0.394	0.768	0.332	0.662
CNN-funnel	**0.479**	0.586	0.391	0.762	0.329	0.663
CNN-hierarchical	0.430	0.611	0.450	0.780	0.411	0.685
UTH-CCB (baseline)	0.440	0.601	**0.480**	**0.787**	**0.438**	0.681

These are the precision (positive predictive value), recall (sensitivity) and F1 metrics for the unseen-disease experiment (i.e. the same experiment described in Table 3). The cells in bold indicate the model which obtained the highest score for the metric

### Results of application on our dataset

Our method produced 651,149 sentences with disease references labelled with our 4-class labelling scheme. This was the largest dataset ever assembled for the task of classifying FP disease references in clinical text, over 100 times larger than the next largest reported in the literature.^5^ Our method was able to incorporate disease references drawn from 1.2 m clinical documents, whereas the combined previous four largest manually labelled studies^5,13,14,18^ drew from a total of 4396 documents (see Table 5).

**Table 5:** Clinical false-positive (FP) dataset sizes reported in the literature

Dataset	Sentence count	Document count
VetCompass (our method)	651,149	1,172,396
VetCompass (Cheng et al.^5^)	6582	1041
BioScope^18^	6383	1953
2010 i2b2/VA^13^	Not reported	871
SemEval-2015^14^	Not reported	531

Our method produced a label indicating if a patient has been diagnosed with the diseases referenced in their clinical notes without the need for an annotator to manually label a sentence. The application of the method to the VetCompass corpus of veterinary clinical notes produced 100 times more sentences for training disease-reference classifiers than the next largest reported dataset (a manually labelled dataset also from the VetCompass corpus). Bioscope had 20,000 sentences in total, but only 6383 came from clinical documents (radiology reports), the remainder were from research papers

The best-performing classifier for both the seen- and unseen-disease experiments was the BILSTM model which obtained the highest AUROC score for all four classes in both experiments bar one; the seen-diseases ‘During window’ class where the BILSTM obtained the second highest score behind CNN-hierarchical (see Tables 1 and 3). The most frequently observed classes in the dataset were ‘Never diagnosed’ and ‘Post-window’ which together compromised 96% of all sentences (see Table 6), so performance in these two classes was especially important. The BILSTM model obtained the highest AUROC score in both these classes in both experiments.

**Table 6:** Class frequency and prevalence percentage for VetCompass primary-care and referral data subsets

Data subset	Never diagnosed	Pre-window	Post window	Inter-window
Primary care	46,018 (50.9%)	8869 (9.8%)	35,254 (39.0%)	201 (0.2%)
Referral	195,763 (34.9%)	13,988 (2.5%)	347,994 (62.1%)	3062 (0.6%)
Total	241,781 (37.1%)	22,857 (0.4%)	383,248 (58.9%)	3263 (0.5%)

Our method was adapted separately to two different subsets of VetCompass data: primary care and referral. Each subset produced a different distribution of class labels

In general, the deeper CNN models (CNN-funnel and CNN-hierarchical) performed better than the shallower CNN-flat in nearly all classes and experiments. This indicated that the hierarchical architectures were better at learning representations of the input sentences than the CNN-flat model which instead focussed on fitting convolutions directly to the word embeddings in the input sentences. CNN-funnel was outperformed by CNN-hierarchical in all classes and for both experiments. This may suggest that it was useful to represent the sentence as a hierarchy of layers, as opposed to just processing it as a series of layers, but further experimentation would be useful to explore the differences further. We found that UTH-CCB was a strong baseline, particularly in the unseen-disease recall metric (see Table 4), where it obtained the highest score of all the models.

Prior work on veterinary clinical data showed that classifiers trained on in-domain data showed decreased performance on out-of-domain data,^5,22^ and we observed the same effect in our experiments for all our models, where performance in the unseen-disease experiments was lower than in the seen-disease experiment. For instance, BILSTM’s AUROC score for the ‘Never diagnosed’ class dropped from 0.898 to 0.730 when evaluated against diseases it was not trained on (compare Table 1 with Table 3).

## Discussion

This study highlights the ability to construct large-scale datasets for classifying disease references in veterinary patients’ notes according to the temporal relation between the disease reference and a relevant diagnosis. We have shown that the BILSTM model obtains the best results for this task, obtaining a higher AUROC score than the baseline UTH-CCB in all classes for both experiments. The other advantage of the neural models was that they required minimal feature engineering, as they worked on word embeddings, whereas UTH-CCB required domain- specific tuning and syntactic dependency parsing which was time-consuming on a dataset of this size. All classifiers were able to fit to data drawn from hundreds of different clinics, including two different market sectors: primary care and referral. This showed that it is possible to get good classification results in this task without the need for expensive manual annotation of disease references.

Our dataset was drawn from the VetCompass corpus, but we needed to adapt our method to two different subsets of VetCompass data: primary care and referral. The main difference between the two subsets is the source of the codes: for the referral subset, the codes were applied in-clinic by the attending clinician, whereas for the primary-care subset, the codes were applied retrospectively by an epidemiologist. We discuss the referral data subset first, since we believe this is the most typical scenario.

The referral subset was constructed from the EMR of patients under referral care at two different referral hospitals: The Royal Veterinary College (RVC) Queen Mother Hospital for Animals (https://www.rvc.ac.uk/small-animal-referrals) and the RVC Equine Referral Hospital (https://www.rvc.ac.uk/equine-vet/hospital-and-specialists). We used the clinical codes which were applied in-clinic by the clinicians responsible for the patients’ care. Coding rates at these clinics were high with almost all patients receiving at least one code per visit, and most patients getting multiple codes per visit. We chose to apply our method using 168 diseases, where we were able to identify disease phrases with relatively good precision (positive predictive value) and recall (sensitivity) for their corresponding clinical codes according to a veterinary named-entity dictionary. Our method for constructing datasets has a weakness in that it is only amenable to phrases which are specific enough to map to a few clinical codes. We found that care must be taken when choosing disease phrase and code pairings. For instance, “mass” maps to hundreds of clinical codes and there is no way of automatically determining which code matches the disease reference. Our method also cannot differentiate amongst the different meanings of polysemous or homonymous words. To take one example, “regurgitation” nearly always referred to food regurgitation, but sometimes it referred to mitral-valve regurgitation (a heart problem). In this case, we paired “regurgitation” with both sets of codes relating to food regurgitation and the heart condition. This meant that the method would label a reference to “regurgitation” as post diagnosis, if it was written in notes after a diagnosis denoted by either set of codes.

We will next discuss how we applied our method to the VetCompass primary-care data subset. Clinical coding rates in primary-care veterinary clinics in the United Kingdom tend to be low: many animals never get any diagnostic codes. Hence, coding in VetCompass is performed retrospectively by clinical veterinary epidemiologists using the vetcompass.org^28^ system. Using vetcompass.org, epidemiologists with a clinical background coded a random sample of patients based on the information in their clinical notes using the veterinary-specific VeNom coding system.^29^ This work was undertaken prior to this research and for the purposes of other epidemiological projects. We combined coded data from 18 separate projects on different diseases, such as demodicosis (a parasitical disease), chronic kidney disease, osteoarthritis and immune-mediated heamolytic anaemia, among others. We used the keywords from the epidemiologists’ keyword searches to identify disease references in the text. The date of diagnosis was decided by the epidemiologist via case review. The criteria of the choice of this date would have been dependent on the study definition criteria chosen by the epidemiologist for their particular study. For instance, one criterion might have been ‘attending vet believed the patient had the disease on this date’. Typically, these dates would have been the date the original attending vet diagnosed the patient. We will explain how our method was applied to this dataset using a concrete example: the VetCompass™ study on demodicosis in dogs.^30^

The epidemiologist curated a list of search terms which picked out references to the disease (examples given in Python regular expression syntax): r’/bdemod[a-z]*/b’,r’/bdemodi[a-z]*/b’,r’/bdemodicosis/b’,r’/bdemodectic/b’. The search terms were matched against the lower-cased clinical notes of 455,557 patients that were included in the epidemiological study. The search terms were chosen to maximise sensitivity and so cover issues like spelling errors or inflected forms insofar as these issues can be handled with wildcard matching. The epidemiologist then read the clinical notes of the subset of patients whose notes contained a match on at least one of the search terms. The epidemiologist determined the diagnostic code and date of diagnosis according to the notes. We built on this earlier work to apply our method: we used the regular expression terms, the labels and the diagnosis dates, and the notes of the 455,557 patients, to construct a set of sentences containing references to demodicosis and labelled them according to our method using the original epidemiologist’s labels and date metadata. This process was repeated for each study (or a set of clinical codes in the case of referral data), and the resultant sentences were combined to form the overall dataset for this work.

Our method requires a hyperparameter to be chosen for the duration of the diagnostic window around the point of diagnosis. We found that a relatively short diagnostic window of 8 h obtained the best classification results on our dataset. The window we chose opened 8 h before the patient was coded and ended at the point of coding. A shorter window was preferable, because it entails fewer sentences getting the inter-window label (0.5%, see Table 6), which means there are fewer disease references whose TP/FP status is ambiguous as defined by our method. The class distributions (see Table 6) for the primary-care dataset show that 9.8% of mentions were written pre-window, and only 0.2% were during the window, suggesting that earlier mentions were mostly occurring on previous visits, leading up the visit that the diagnosis was made on. For referral cases, only 2.5% of sentences were pre-window; this could indicate that patients had fewer visits to the referral centres, or that they were diagnosed faster.

The number of primary-care and referral patients differed markedly in the wider VetCompass corpus at the time of study: 5.9 m primary-care patients vs. 174 k referral patients. However, our method yielded only 90 k labelled sentences for the primary-care patients and 561 k for the referral patients. This was for two reasons. Firstly, although there were fewer patients in total in the referral dataset, there was a much higher proportion of coded patients. Secondly, we could use EMR from the referral data subset which contained disease references but which were uncoded (these were labelled as Never diagnosed). This is because in the referral setting, a clinician wrote the patient’s notes and decided not to code the patient; an uncoded patient in the referral setting indicated an absence of disease. However, we could only use a primary-care patient’s EMR if the reviewing epidemiologist coded the patient as either having the disease or declared them free of the disease; an uncoded patient in the primary-care setting indicated only that the epidemiologist had not reviewed the patient’s notes.

## Methods

### Dataset preparation

Firstly, we split the clinical notes taken from the EMR into sentences and we discarded any sentences that didn’t contain references to diseases of interest. Classification of disease references was at the reference level (rather than the sentence level), so sentences were duplicated as required for each disease reference. The resultant sentences were *X*. Our labels *Y* were defined according to the previously discussed sentence-labelling scheme.

Our method required the following:

- Longitudinal EMR with clinical documents and clinical codes. Both must be time-stamped so that they can be ordered. The codes don’t have to follow a strict or consistent coding guideline.
- A method of pairing disease references in the text with an equivalence set of clinical codes, indicating a diagnosis of the disease.
- A hyperparameter value to be chosen for the duration of the diagnostic window.

The first requirement is satisfied by most EMR. For the second requirement, multiple approaches are viable: a dictionary or named-entity recognition system,^31^ or simply write a list of disease references paired with diagnostic codes. We experimented with two different methods, one for primary-care data, and one for referral data, which we discuss in more detail in the Discussion section. The third requirement is a hyperparameter of our method (indicatively, windows of a few hours worked better on our data).

### Sentence vectorisation

We now show how we vectorised the raw sentences and constructed our classifier. *X*_*i*_ is a matrix representing a sentence which references a disease of interest. *X*_*ij*_ is a vector which represents the *j*th token in sentence *X*_*i*_. Our token vectors were trained in an unsupervised fashion using a skip-gram word2vec model.^32^ We concatenated a single feature with the word2vec token representation: since sentences can reference multiple diseases but we only want to classify one, we added a single binary feature to all tokens *X*_*ij*_, such that the feature was 1 when the token formed part of the disease reference we want to classify, and 0 otherwise. For example, in the following sentence, the disease reference is in bold and has its token’s binary feature set to 1, all other tokens are set to 0: “discussed possible causes such as thyroid diabetes and renal disease.” This feature gives the classifier the potential to give different labels for the same input token sequence, as without this feature, there is no way to indicate to the classifier which disease reference in the sentence we want it to classify. The input to the classifiers were the sentences *X* and the output was one of the four class labels for each input sentence.

We trained the word2vec token representations on a sample drawn from the wider VetCompass™ corpus (55 million clinical documents, 2.3 billion tokens), not just from the tokens in *X*. The neural models required fixed-length inputs and so cannot work on the variable-length sentences observed in the data. The number of tokens in a sentence $|X_i|$ was a hyperparameter of our method; we chose 300 tokens. If an input sentence didn’t contain 300 tokens, we truncated or zero-padded it, so that all our sentences were of the same length.

### Limitations and future work

One limitation of the approach described is that the method used a fixed-size diagnostic window of 8 h. A future work might investigate a dynamic window size, which may better capture variability in the underlying data. It would be interesting to determine if classification performance increased if each disease had its own window size. Also, for some patients, visits take place over several days. We suspect that these patients tend to get coded at the point of discharge. A subsequent experiment could set the window size to the scope of the visit which received the code, which would mean that disease references during a single visit would receive a consistent label. Finally, the writing style differed between referral centres and primary-care clinics; referral notes tend to contain grammatical sentences and primary-care notes tend to contain more short-hand or bullet points. We don’t know if this was a factor in classification accuracy and a future dedicated study could investigate this.

We have shown that it is possible to create a large corpora of example sentences for training FP classifiers from EMR that were not annotated for this purpose. We have also demonstrated that a number of different models can be trained on this corpus. The best-performing model was the BILSTM model. Ethics approval was given by the Clinical Research and Ethical Review Board at the Royal Veterinary College under URN 2015 1369.

### Reporting summary

Further information on research design is available in the Nature Research Reporting Summary linked to this article.

## Supplementary information

reporting summary

## Data Availability

The datasets in this study are not publicly available due to privacy and data protection concerns, but are available with restrictions from the corresponding author.

## Supplementary information

**Supplementary Information** accompanies the paper on the *npj Digital Medicine* website (10.1038/s41746-019-0108-y).

## Code availability

Our code is available at https://github.com/RoyalVeterinaryCollege/detecting_false_positives.
